# Trends and Applications of Omics Technologies to Functional Characterisation of Enzymes and Protein Metabolites Produced by Fungi

**DOI:** 10.3390/jof7090700

**Published:** 2021-08-27

**Authors:** Grace N. Ijoma, Sylvie M. Heri, Tonderayi S. Matambo, Memory Tekere

**Affiliations:** 1 Institute for the Development of Energy for African Sustainability (IDEAS), College of Science, Engineering and Technology, University of South Africa, P.O. Box 392, UNISA, Pretoria 0001, South Africa; sylvieheri92@gmail.com (S.M.H.); matamts@unisa.ac.za (T.S.M.); 2 Department of Environmental Science, College of Agricultural and Environmental Science, University of South Africa, P.O. Box 392, UNISA, Pretoria 0001, South Africa; tekerm@unisa.ac.za

**Keywords:** fungi, omics, lignin modifying enzymes, bioinformatics

## Abstract

Identifying and adopting industrial applications for proteins and enzymes derived from fungi strains have been at the focal point of several studies in recent times. To facilitate such studies, it is necessary that advancements and innovation in mycological and molecular characterisation are concomitant. This review aims to provide a detailed overview of the necessary steps employed in both qualitative and quantitative research using the omics technologies that are pertinent to fungi characterisation. This stems from the understanding that data provided from the functional characterisation of fungi and their metabolites is important towards the techno-economic feasibility of large-scale production of biological products. The review further describes how the functional gaps left by genomics, internal transcribe spacer (ITS) regions are addressed by transcriptomics and the various techniques and platforms utilised, including quantitive reverse transcription polymerase chain reaction (RT-qPCR), hybridisation techniques, and RNA-seq, and the insights such data provide on the effect of environmental changes on fungal enzyme production from an expressional standpoint. The review also offers information on the many available bioinformatics tools of analysis necessary for the analysis of the overwhelming data synonymous with the omics approach to fungal characterisation.

## 1. Introduction

The use of fungal biological products predates this modern era; consistently, fungi have been used as a source of food, food additives, hallucinogens, pharmaceuticals (antibiotics, hormones, and immunological adjuvants), citric acid, specialty chemicals production, and derived enzymes have been used in a variety of bioprocesses including textile bleaching, alcohol fermentation, and environmental pollution remediation [1,2,3,4]. However, the isolation and identification of a desired species of fungi and its functional characterisation to determine the potential biological product that it will contribute towards domestic and commercial exploits are prerequisite stages for the commencement of any application. The precise identification of a fungal strain is crucial, especially when we consider that visual misidentification by unsuspecting individuals and resultant ingestion have often led to grave consequences, including fatalities [5,6]. Moreover, the pertinent role played by members of the fungi kingdom as major decomposers and recyclers in nature and their ubiquity in both terrestrial and aquatic environments has motivated investigations that culminate in the accepted importance of their degrading enzymes in several industries. Some fungi have been isolated and known to thrive in very extreme conditions such as arid desert conditions, low pH and high metal concentrations such as is present in some soils, mine tailings and acid mine drainages, and even in high-salt concentrations as found in marine ecosystems [7,8,9,10]. In the quest for survival by the fungi, these extreme environments have facilitated the evolution of unique enzymes that find applications in several industrial and environmental exploits. The most important enzymes studied to date are those produced by the species of Ascomycetes, Basidiomycetes, and Deuteromycetes, particularly the lignin-modifying enzymes (LMEs) such as cellulases, laccases, and peroxidases that have also proved very useful in the degradation of xenobiotic compounds due to their multi-specific activities [11,12,13].

In the past, research on fungal characterisation relied mostly on the classical mycological and microbiological approaches of bioprospecting which involved isolating wild fungal species of interest from their natural environments, cultivation under growth conditions that strived to simulate favourable conditions for proliferation using selective growth media on plates and sometimes cultivation in submerged cultures, under aseptic culture conditions [14,15,16]. However, even with advancements, not all taxa have been successfully cultured on media, and some fungi have demonstrated recalcitrance to cultivation on most current commercially available media. More recently, there have even been attempts to overcome this challenge with the use of laser-printing technology [17]. The earliest studies involving fungi used adaptations of the laser-induced forward transfer (LIFT), such as absorbing-film-assisted, laser-induced forward transfer (AFA-LIFT) for the fungus (*Trichoderma*). The LIFT enables the rapid but controlled transfer of minute quantities of biological samples to different substrates thinly coated to a metal surface plate with other materials such as silica. However, AFA-LIFT especially ensures this thin layer is protected from the laser irradiation by a metal film capable of absorbing the laser energy and conversion to kinetic energy; this energy actually stimulates rapid germination and/or growth of conidia as was observed in *T. longibrachiatum* with germination observed within a 20 h incubation period [18,19,20,21].

In general, growth and isolation are typically achieved using solid-surface culture techniques; fungal growth in liquid media is also possible, especially for the purpose of enrichment to maximise the proliferation of mycelia of a desired fungus [22]. However, submerged cultures cultivation is challenged by oxygen levels constraints for these aerobic eukaryotic cells as the mycelia will tend to grow on the liquid–air interface. Nonetheless, the latter cultivation approach has undergone several adaptations, including pseudo-submerged culture conditions, as the growth in liquid media appears to favour the collection of the extracellular enzymes produced by fungi [23].

**Figure 1:**
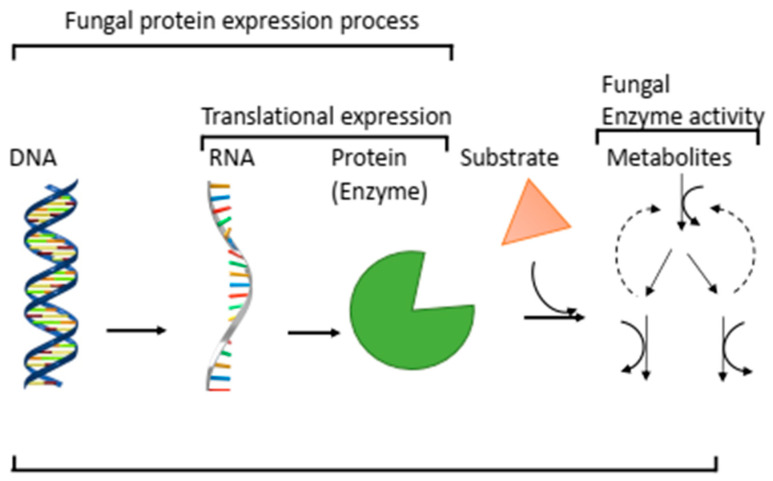
Fungal protein (enzyme) expression process.

Although the process of obtaining axenic cultures of fungi is tedious and requires several sub-culturing and strain transfers on selective media, it is unavoidable in the quest to derive biological products from a fungal strain [24]. Moreover, pure plate cultivation ensures storage and reproducibility. In the past, identification was based on observable traits, and characterisation employed morphological variations that relied on nuanced visualisation facilitated by staining techniques and microscopy, for example, light, electron, fluorescence, phase contrast, etc. [25,26]. Morphological characterisation depends greatly on comparative analysis with previously documented fungal features [27]. Even with advances in microscopy and the additional computational analyses that have improved its accuracy, morphological characterisation is still considered an imprecise method, especially when applied for differentiating members of the same genus and when, at mycelial stages, distinct features have not developed. The reliance of microscopy on an individual’s expertise further increases biases in identification [28]. Therefore, beyond phenotype-based identification, genotype studies appear to provide the best option in non-discriminatory characterisation. This review provides a synthesis of information on progress made thus far in integrating the omics approach in fungal investigation and functional characterisation of economically significant enzymes in the hope of bringing attention and encouraging routine use of these frameworks in research to boost the lagging database for fungi-derived biological products. There is a general acceptance that any progress in fungi-derived product upscaling and increased industrial product volumes can only be the result of our clear understanding and elucidation of fungal responses to environmental changes, their molecular expression patterns and mechanisms of metabolite productions and adaptations. Such knowledge will be concomitant to advances in the field of mycology and industrial applications. Moreover, it will be associated with the ease in accessibility and cheap application of enzymes and other protein metabolites employed in industrial and environmental processes. Additionally, the application of these enzymes will potentially reduce effluent production owing to its specificity in chemical reactions, and it will serve as an environmental pollution mitigation strategy. Therefore, it becomes necessary to routinely re-evaluate the status of advances made in fungal characterisation to allow researchers to identify gaps requiring improvements. The omics approach, as will be discussed, encompasses metagenomics, genomics, transcriptomics, and proteomics. Figure 1 presents an overview of the protein (enzyme) expression process and the connection to the omics approach in functional characterisation. Research thus far has been sparse, in which omics technologies have been comprehensively used in the characterisation of novel fungal enzymes. In a recent publication, Oates et al. [29] used a multi-omics approach to identify a number of potentially important CAZymes from *P. putredinis* NO1. A potential industrially relevant extracellular oxidase capable of cleaving β-ether linkages and releasing tricin from monocot lignin was also identified. Brenelli et al. [30] used a similar approach to identify multiple redox-active enzymes produced by the marine fungus *Peniophora* sp. CBMAI 1063. Using a combination of genome and secretome analyses, the authors discovered valuable extracellular CAZymes, especially lignin and polyphenols-degrading enzymes. It is hoped that this review will draw attention to this gap in research and provide insights on a systematic approach to fungal protein metabolite characterisation.

## 2. Relevant Metabolites Produced by Fungi

**Figure 2:**
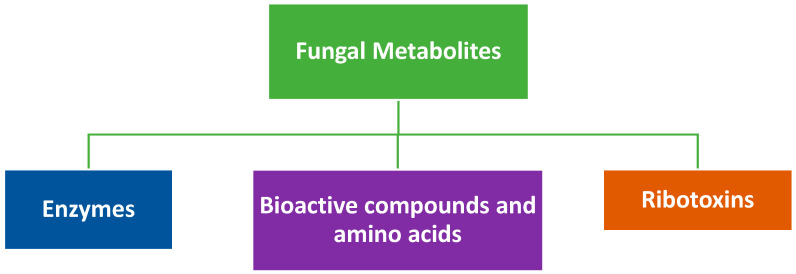
Relevant fungal metabolites.

Fungi produce both primary and secondary metabolites of medical and economic significance. Pertinent to the review are three major types of metabolites (see Figure 2): the enzymes, bioactive compounds (including amino acids), and fungal ribotoxins. Although it is estimated that at least 5.1 million species of fungi exist, the Basidiomycota and Ascomycota phyla appear to have predominance and include several wood- and litter-decomposing fungi [31]. Moreover, these two phyla have thus far produced the most impactful metabolites, including enzymes that have gained industrial importance. They have shown demonstrable application in carbon recycling and therefore bioremediation, as well as usefulness in food, feed, and pharmaceutical industries [32,33,34,35,36]. The enzymes produced by fungi are unique because of their broad substrate specificity as well as their strong oxidative and hydrolytic abilities, and of utmost importance is the ease of collection since most of them are expressed extracellularly. Table 1 shows some important examples of industrially relevant fungal enzymes and the aspects of industry in which they have found application. Several authors in recent times have reviewed the applications of these enzymes [37,38,39].

There are additional enzymes that have been recently elucidated that find application in the bioremediation of recalcitrant pollutants as well as heterocyclic substrates, including aromatic hydrocarbons, halogenated biphenyl esters, and chlorinated benzenes [40]. These two newly elucidated enzymes are chloroperoxidase and the so-called aromatic or unspecific peroxygenases (APO/UPO) and are classified as either heme-thiolate peroxidases or peroxygenases with capabilities to incorporate an oxygen molecule from their hydrogen peroxide acceptor into their substrate, thereby demonstrating peroxygenase activity [41]. Moreover, there has been the identification of dye-decolourising peroxidases (DyP) in the heme peroxidase class with a preference for the oxidation of anthraquinone dyes. These enzymes show high catalytic efficiency and, in the future, will most likely play a very important role in the decolourisation of synthetic dyes [42,43].

Several secondary metabolites are produced by fungi, often referred to as bioactive compounds; most of these metabolites are associated with endophytic fungi, a group of fungi closely associated with plants. These bioactive compounds have demonstrated efficacy as antibiotics and anticancer drugs and include Penicillenols, Taxol, Clavatol, Sordaricin, Jesteron, and Javancin. Bioprospecting of these groups of fungi offers great potential for investigation and drug discovery as it is believed that only a small fraction of these compounds has been identified to date. Recently, Hoeksma et al. [44] used a combination of mycology, embryology, and chemistry to test and identify biologically active secondary metabolites from over 10,000 species of fungi using developing zebrafish embryos. The authors successfully identified 34 active compounds consisting of both therapeutically known and unexplored compounds. Molecular characterisation approaches have also recently been applied for the screening of bioactive compounds from endophytic fungi [45].

Furthermore, fungi produce a number of free amino acids that contribute nutritional value and unique tastes to food preparations. Previously, Tseng et al. [46] identified free amino acids as some of the non-volatile flavour components of the medicinal mushroom *Ganoderma tsugae*. A total of 17 amino acids were found to be present. Recently, Tagkouli et al. [47] cultivated fungi on agricultural by-products and used this to specifically assess the free amino acids profile of *Pleurotus* mushrooms. The authors identified a total of 22 free amino acids, including all the essential ones. The work by Landi et al. [48] on the Pioppino mushroom (*Cyclocybe cylindracea*) also illustrates the presence of these important products in basidiomycetes as their content analysis of this mushroom revealed the presence of various amino acids and their antioxidant activity.

**Table 1:** Economically significant enzymes produced by basidiomycetes and ascomycetes.

Class of Enzyme	Sub-Category	Enzyme	Industrial Applications	References
**Oxidoreductases**	**Heme-containing** **peroxidases and peroxygenases**	Lignin peroxidase	Production of vanillin, textile dye degradation and bleaching, xenobiotic compounds degradation	[37]
		Manganese peroxidase	Bioethanol production, dye decolourisation, degradation of rubber, distillery waste treatment	[37,39]
		Versatile peroxidase	Novel delignification strategies	[38]
		Chloroperoxidase	Halogenation and oxygenation of organic compounds, lignin cleavage	[41,49]
		Aromatic/Unspecific peroxygenases	Bioremediation of recalcitrant pollutants such as polycyclic aromatic hydrocarbons, halogenated biphenyl esters, and chlorinated benzenes	[40]
		Dye-decolourising peroxidases	Decolourisation of synthetic dyes	[42,43]
	**Flavin-containing** **oxidases and** **dehydrogenases**	Aryl-alcohol oxidase	Synthesis of high value-added chemicals, flavours, production of bio-based polymer precursors, dye decolourisation, pulp bleaching	[50,51]
	**Copper-containing** **oxidases and** **monooxygenases**	Laccase	Detoxifier in industries such as pulp and paper, textile, and petrochemical Heavy metal precipitation, biosensor production, food processing, drug preparation	[52,53,54]
**Hydrolases**	**Proteases**	Alkaline, acid, and neutral proteases	Fabrication of laundry detergents, beer haze clarification, brewing, cheese making, meat tenderisation, synthesis of bioactive peptides	[33,34]
	**Glycoside** **hydrolase**	Cellulases: endoglucanases, exocellulases, and processive endoglucanases	Juice extraction and clarification, supplementation of livestock, biofuel production, laundry detergent production	[35]
	**Xylanase**	Hemicellulases, endo-1,4-β-D-xylanases, β-D-xylosidases, α-glucuronidase acetylxylan esterase, α-L-arabinofuranosidases, p-coumaric esterase, and ferulic acid esterase	Juice clarification, paper bleaching, and increasing the nutritional value of animal feeds.	[36,55]
	**Amylase**	α-amylase, β-amylase, and glucoamylase	Bread making, detergent manufacture, treatment of digestive disorders	[56]
	**Lipase**	Triacylglycerolacyl hydrolases	Production of detergents and cosmetic products, synthesis of biopolymers and biodiesel, manufacturing of food products	[36]

In recent times, attention has been focused on the highly toxic extracellular ribonucleases produced by fungi because they have previously demonstrated antitumor and immunotoxin effects [57], making them useful in cancer therapy and immunotoxin synthesis. Ribotoxins exert ribonucleolytic activity on the larger molecule of RNA of the ribosome, which results in the cessation of protein synthesis and cell death through apoptosis [58]. Olombrada et al. [59] reviewed the potential biotechnological applications of these highly specific enzymes. Research completed by Li and Xia [60] showed the efficacy of ribotoxins as pest control agents either alone or conjugated with other compounds. This was used in experiments involving a recombinant form of the insecticidal ribotoxin hirsutellin A (HtA) produced from *Pichia pastoris*. The insecticide formulation showed dose-dependent cytotoxicity to Sf9 insect cells and insecticidal activity against *Galleria mellonella* larvae.

Recently, a novel family of proteins similar to ribotoxins has been identified and characterised. Landi and colleagues first characterised and purified Ageritin, the first basidiomycete-produced ribotoxin-like protein capable of inhibiting protein synthesis in vitro and releasing the α-fragment when incubated with ribosomes [61]. More recently, the same researchers identified a second ribotoxin-like protein called Ostreatin [62]. Although functionally similar to ribotoxins, these enzymes show no homology with ribotoxins from the ascomycetes family. They show more similarity to members of the ribonuclease T1 family, however. Ragucci et al. [63] recently reviewed Ageritin, focusing on its structural, biological, antipathogenic, and enzymatic characteristics. The authors also discussed some of the possible biotechnological applications of Ageritin, including its use as an immunotoxin and pest control agent, which could be achieved through gene transfer in plant cells.

## 3. Advances in Molecular Characterisation of Fungi

The study and application of molecular biology to fungi characterisation has rapidly gained popularity as a gold standard approach and is entrenched now as validation for most morphological characterisation [64]. Its routine adoption to the taxonomic identification of pathogenic fungi has greatly assisted in the resolution of previously unidentified fungi to species level [26,65,66]. The process begins with genomic extraction techniques, sequencing which employs specific DNA markers that allow for identification to species level; further bioinformatics analyses facilitate phylogeny establishment.

Developed in 1977, Sanger sequencing is the current standard molecular tool for identification [67]. Sanger sequencing is made possible through polymerase chain reaction (PCR), a molecular technique that enables the synthesis of a complementary DNA strand from a template using the enzyme *taq* polymerase [68,69]. In 1990, a ground-breaking advancement in fungal molecular characterisation was introduced with the identification of fungal nuclear ribosomal RNA (rRNA) operon primers [70]. The DNA sequences associated with the large ribosomal subunit (nrLSU-26S or 28S), the small ribosomal subunit (nrSSU-18S), and the whole internal transcribed spacer region (ITS1, 5.8S, ITS2; 650-900 bp) have since become the target region of fungal identification by the Sanger sequencing approach [64]. Different evolution rates have been observed in the ITS region, resulting in diverse levels of variation from one organism to the next. As the fastest evolving and most variable segment, the ITS region has become the golden standard for fungal identification using ITS1 and ITS4 primers [64,71,72,73]. The ITS1 and ITS4 gained popularity because they are capable of differentially identifying to significant extents members of the Ascomycetes and Basidiomycetes class of fungi. Molecular characterisation also owes its success to the rapid advancement in bioinformatics tools and the continuous addition of new species and strains to different databases that enable identification. Molecular characterisation involves the initial amplification of a conserved DNA region using a genomic DNA template and specific primers that target specific regions. Amplification is then terminated using di-deoxynucleotides. The derived sequences are matched to other previously submitted sequences in a comprehensive database. These comparisons characterise and identify through the matching of sequences to the closest relatives on the database by allocating a value range of between 0 and 100 for percentage similarities, the latter indicating an exact match [67,74]. Databases such as e-Fungi, NCBI-BLAST, FungiDB, and UNITE serve as repositories for sequences and are also crucial to molecular characterisation as it is through them that identification can be precisely achieved [75,76,77]. Nilsson et al. [78] as well as Kõljalg et al. [79] describe UNITE, a database designed for fungi molecular identification curating all public fungal sequences as well as those that have not successfully been assigned to any taxonomic lineage beyond phylum. The latter groups are assigned a unique digital object identifier (DOI) in the interim. Loeffler et al. [80] discuss the importance of reference genomes to metagenomics studies but pertinently warn of the poor integration of new sequences into the existing database as well as the cooperative interactions between different databases with the resulting lack of a credibly comprehensive database despite progress in sequence curation. The direct implication is the doubtful identification of any fungi that are analysed using only one reference database [81]. This challenge significantly impacts progress in metagenomic analysis as it hinders the pace of identification and adds the cumbersome process of further analysis on several databases to ensure that accuracy in identification is achieved. The consensus solution will be to open dialogue between database creators and seek a partnership that allows the merging of most platforms to reduce the overlap created and prevent the ‘re-invention of the wheel’, synonymous with a lack of cooperation amongst developers. This cohesion will reduce discrepancies in identification between multiple platforms and databases and accelerate progress in functional genomics.

## 4. Transcriptomics in Fungi Functional Characterisation

Genomic sequences provide, to a certain extent, sufficient information that facilitates the molecular characterisation of fungi. However, it is by no means adequate in giving insights into an organism’s physiology because the sequence data elucidate all genes present; however, these genes are usually not expressed at the same time or in the same manner [82]. Changes in environmental conditions significantly influence gene expression and metabolites production by causing epigenetic modifications such as DNA methylation and chromatin re-modelling [83]. This implies that to truly understand metabolite production, it is important to look in-depth at the succession of events that occur during gene expression under normal circumstances and make comparisons against varied changes in conditions. This aspect of research is referred to as transcriptomics and has also evolved over time in response to technological advances for fungal functional genomic characterisation.

The central dogma of molecular biology is premised on the transcription of genes into messenger RNA (mRNA) and the translation of the latter into peptides; it also integrates the study of the post-translational processes and the organisation of polypeptide chains into functional proteins [84,85]. This study is important in connecting genes to morphological and physiological characteristics. More recently, the focus is firmer on gene expression going from the genome to the transcriptome [86]. The field of transcriptomics bridges the gap between genes and proteins [87] and has made it possible to monitor and measure gene expression in different cells and tissues as well as the responses to different conditions at different time points. More importantly, with eukaryotic cells and their pre-disposition towards expression being directed by a complex array of genes, but also in some other instances, expression may be the consequence of single gene’s activity, while others can even be attributed to the activity of a cluster of genes [88]. Advances in our understanding of transcriptomics have enabled the identification of many previously unannotated genes, such as witnessed in the recent work of Noriega et al. [89], where transcriptomics provided insights to genes crucial to different developmental stages in the coffee berry borer *Hypothenemus hampei*. There are similarities in the approaches used by Noriega and colleagues to that employed by Kim et al. [90] in the identification of genes associated with a particular phenotype; in their research, the focus was on identifying genes responsible for the display of yellow colouration on leaves obtained after the γ-ray-based mutagenesis of a *Cymbidium* orchid. They utilised the Kyoto Encyclopedia of Genes and Genomes database to assign a total of 144,918 unigenes derived from over 25 million reads into 22 metabolic pathways. Remarkably, the RNA sequencing analysis identified 2267 differentially expressed genes between wild-type and mutant *Cymbidium* sp. In summary, they were able to pinpoint the alteration of chlorophyll metabolism to seven genes also observed to be involved in ion transport as well as chlorophyllase-2 production. This implied a possible link between leaf colouration in *Cymbidium* orchids and combined alterations in chlorophyll metabolism and ion transport.

Transcriptomics has also contributed to our understanding of white-rot fungi and the process of LMEs production. Although it is still sparsely utilised in fungi research, techniques such as quantitative reverse transcription PCR (RT-qPCR) have been found to be very useful in identifying and quantifying mRNA at any given time in biological systems [91] and can be integrated into fungal differential expression studies. It should be noted that in comparison to other PCR techniques, RT-qPCR gives accurate quantification of mRNA due to its ability to quantify in real-time. Therefore, it is very useful in investigations of gene expression resulting from different treatments. Moreover, it has other advantages, including the absence of a need for amplification, an exercise that tends to introduce bias; it also avails the user a mid-throughput analysis and, to an extent, ease of automation that allows for the processing of a relatively significant number of samples [92,93]. RT-qPCR stabilises a target mRNA through its transcription into complementary DNA (cDNA) while amplification is ongoing; there is an associated emission of fluorescence which enables real-time quantification [94]. Fernández-Fueyo et al. [95] used RT-qPCR to investigate the effect of environmental parameters of temperature and pH on gene expression and regulation in *Pleurotus ostreatus*. By monitoring gene expression under various conditions, the authors were able to present certain predictions on the correlation between temperature and pH in gene regulation. Previously, RT-qPCR was used to identify the peroxidase genes *lip* and *mnp* from soil fungi [96] and in studies involving peroxidase genes in ectomycorrhizal fungi [97], as well as the expressional profile of *Pleurotus ostreatus* laccase genes [98] and many more. More recently, Vasina et al. [99] used similar tools in the quantification of the expressional patterns of 18 peroxidase genes encoding class II peroxidases in *Trametes hirsuta*. Moreover, the characterisation of this multigene family enabled the design of specific primers that were used for further studies.

Although the RT-qPCR is considered fast and effective, its limitation remains its mid-throughput feature which implies that it can only process a few genes at a time. This can be a constraint, especially impactful when studying eukaryotic organisms and fungi with their tendency to have multiple gene involvement in the production of a given compound. Therefore, it limits the depth of analysis in such instances [100,101]. Moreover, a further challenge in employing RT-qPCR in fungal investigations is with the choice of a correct reference gene, which is sometimes hampered by the sharp impact that environmental/external parameters and growth conditions tend to have on gene expression [102]. This has motivated the introduction of novel, high-throughput technologies in order to ameliorate these limitations [87,103]. Hybridisation techniques such as microarrays were able to partially address these issues by increasing the amounts of genes to be analysed per run. However, prior knowledge of these genes is still required to develop complementary probes [87]. It would appear that most of the limitations highlighted previously in this review were addressed in the design and application of the RNA sequencing (RNA-seq) platform development; of most importance is the ability to analyse thousands of known or unknown genes at once without the need for a reference gene [104,105,106]. Moreover, the RNA-seq platforms have the ability to integrate analysis of post-transcriptional modifications, which is important in eukaryotic cell studies and specifically fungi, where gene fusion, alternative gene spliced transcripts and mutations/single nucleotide polymorphisms (SNPs), and changes in gene expression may occur over time. RNA sequencing platforms currently use the next-generation sequencing principle to obtain the whole transcriptome profile (type and quantity) of a cell, tissue, organ, or entire organism [107]. Although modifications have been made since its introduction, all RNA-seq platforms basically use the same principles, differing only in terms of read lengths, throughput, error, and price. Different authors have reviewed and compared these platforms for various applications [107,108,109,110]. Ravichandran et al. [111] used RNA-seq on an Illumina platform to obtain insight on the degradative ability of a white-rot fungus based on the expression of genes for these degrading enzymes. Henske et al. [112] used RNA-seq to investigate the differential expression of LMEs in the presence of different substrates. Additionally, Ma et al. [113] previously used the same tool to study fungal metabolic regulation by identifying all genes under the regulation of a given transcription factor, such as the *Xyr* factor presumed to be responsible for carbohydrate metabolism.

In as much as there have been some studies that have helped to deepen our understanding of the expressional patterns followed by fungi, especially with regard to LMEs, several aspects of functionality are yet to be elucidated. Most of these studies point to a need for continuation in research and in-depth studies as many more questions are raised when attempts are made that seek to probe previously held assertions [114]. It is without a doubt that these questions around expressions and translation patterns need investigations especially motivated by the potential applications that these enzymes produced by fungi represent to several industrial and environmental bioremediation pursuits. For example, Korripally et al. [115] used RNA-seq to study the regulation of LMEs gene expression. By applying whole transcriptome shotgun sequencing, they observed an increase of up to four-fold from an initial 356 genes at the inception of ligninolytic enzyme production. This link presents questions on what the possible trigger in terms of external conditions could have been that caused this sharp increase. This is further emphasised because, in the same study, they made further observations that there were at least 356 up-regulated genes and that 165 were of unknown functions. Similar thought-provoking results have been observed in other studies [116,117], which indicate the need for more transcriptomics studies in order to bring better insight to this area of research and possibly serve as a starting point for the elucidation of LMEs gene expression, as it tends to have a direct impact on the amount of enzyme produced.

### Bioinformatics in Fungi Transcriptomics Studies

The evolution in transcriptomics studies and the general acceptance of RNA-seq platforms as standard for transcriptomics-based studies resulted in a change in the approach to data analysis owing to the copious amounts of data it generates. This has motivated a transition to the application of complex bioinformatics and computational tools to enable a clearer interpretation of data, accuracy, and reproducibility of results. These data analyses involve three major steps, namely: quality control of the raw reads, mapping and alignment, and quantification of reads. Similarly, further investigative research on differential expression analysis also involves each of the steps identified and several other bioinformatics tools [87,118]. This stage-gated approach in reviewing data is premised on the susceptibility of the huge amount of complex data to many variations that may arise from technical and random sources [119]. Therefore, the quality control of raw data is especially critical in ensuring the accuracy of analysis, primarily focused on tackling likely biases introduced from nucleotide composition, for example, GC-content that tends to affect differential expression analysis [120]. This problem is usually overcome with normalisation to guarantee accuracy in the inference of expression levels and other analyses, but if this is not considered for prior treatment before analysis, such an anomaly will bias final results. To date, various bioinformatics tools have been developed for RNA-seq quality control (QC). In their work, Hernández-Domínguez et al. [118] present FastQC as one of the most popular QC tools for the Illumina platform. FastQC reports on the quality of data based on reads or sequences run on its platform; it also provides the proportion of each nucleotide base in the reads. The applications and efficiency of FastQC were also reviewed by Qi et al. [121]. Other QC tools, including FASTX-Toolkit, QC-Chain, and NGS QC Toolkit, were discussed by Zhou et al. [122], who also introduced RNA-QC-chain, a novel comprehensive tool for QC which comes with the advantages of trimming, automatic rRNA detection, and contaminating species identification. Quality control not only takes place at the beginning of data analysis but also precedes each step of further data analysis.

Initial quality control is followed by mapping and alignment, where all reads are located either with respect to a reference genome or using de novo assembly. In their work, Schaarschmidt et al. [123] evaluate seven different mapping tools (bwa, CLC Genomics Workbench, HISAT2, kallisto, RSEM, salmon, and STAR) using experimental data from *Arabidopsis thaliana*. Interestingly, similar results are obtained, with all of them showing high reproducibility. Hernández-Domínguez et al. [118] describe three strategies that could be followed during the mapping process. When the goal is to identify new transcripts, reads are aligned with gaps to a reference genome. Tools such as STAR have been found to be best adapted for this type of mapping as they can map spliced sequences of any length [124]. When new transcripts are not the query for analysis, reads are aligned to the reference genome without gaps using tools such as RSEM. In other cases, where a reference genome is not available, reads are used for de novo assembly. Previously, Haas et al. [125] described Trinity, one of the most popular tools used for de novo assembly. Trinity is an ‘assembly-first’ tool for transcriptome reconstruction consisting of three modules, namely, Inchworm, Chrysalis, and Butterfly. Trinity is able to assemble transcriptomes by separating the data into many de Bruijn graphs that are processed separately before using parallel computing to reconstruct the transcriptome [126].

Transcript quantification is an important step in data analysis as it provides the important quantitative aspect to RNA-seq applications which allows comparison between different expression points and provides insights as to when the organisms begin to respond to externalities. Jin et al. [127] evaluated different quantification methods using tools such as TopHat, RSEM, HTSEq, and featureCounts. The authors also differentiated between alignment-based and alignment-free methods depending on the presence or absence of a reference genome. Another important step in transcript quantification is the previously mentioned normalisation of data to remove the influence of all possible biases. Normalised measures such as RPKM (reads per kilobase of exon model per million reads), FPKM (fragments per kilobase of exon model per million mapped reads), and TPM (transcripts per million) are then used to report expression values [128]. Based on the obtained data, differential expression analysis can then be carried out by comparing values from different samples. Various tools are also available for differential expression, including DESeq, Cufflinks, PoissonSeq, UpperQuartile, etc. [107,128]. Although most currently used tools have been found to be very effective for differential expression, Assefa et al. [129] reported low performance when assessing differential expression of long non-coding RNAs (lncRNAs) using 25 different pipelines. The authors correlated this with sample levels and variability, as lncRNAs are expressed at low levels and are quite variable. For fungal specific studies, Wang et al. [130] described a workflow for differential expression in fungal species using the Bioconductor package DESeq2. In recent work, Pawlik et al. [131] used RNA-seq to study differential expression in the fungus *Cerrena unicolor* FCL139 when grown under different lighting conditions. Mapping was completed using a reference genome, and the DESeq 2 package was also used to identify the differentially expressed genes.

## 5. Proteomics in Fungi Translational Characterisation

Translation of mRNA into functional proteins is the second step of the central dogma; it is considered a precursor to metabolomics studies and most cellular biochemical activities. It is also where products of economic significance are derived. Depending on the complexity and size of the organisms’ protein; the final product can be simple or complex, with the latter requiring a further post-translational modification (PTM) step of folding at the cellular or intercellular levels; without this crucial refinement, the long peptide chains that spool out during translation may not gain functionality or specificity [132]. Proteomics looks at the identification and quantification of all proteins present in a given cell, tissue, or organism [133,134]. The study of protein expression, modification, structure, and function by means of proteomics has brought progress in various fields of science and technology because proteins are usually the end-products of gene expression and the sought-after bioproducts. Amiri-Dashatan et al. [135] reviewed the application of proteomics to food technology, biomarker, and drug target identification. An in-depth review of the applications of proteomics in pharmaceuticals was also executed by Yokota [136], including the use of proteomics to study expression profiling, protein–protein interactions, and post-translational modifications. Other authors have focused their proteomics studies on fungi specifically in order to identify potential vaccines and drug targets [137,138,139]. In some of the research studies, the authors used quantitative Mass Spectrometry—Elevated Collision Energy to identify fungal proteins with no significant homology with human ones with the goal to ascertain their feasibility as vaccine candidates. Importantly, Ball et al. [140] reviewed advancements in MS-based proteomics as it relates to fungal pathogenesis and interactions between these fungi and the host. Even though the focus was on medicine and pharmacology, this work provides a benchmark for studies in industrial and environmental biotechnology.

### 5.1. Protein Production and Analysis

Eukaryotes, in general, have more complex machinery involved in protein production as compared to bacteria. Fungi proteins undergo post-translational modifications (PTMs) involving phosphorylation, glycosylation, acetylation, and many other processes to ensure functionality. PTMs take the form of covalent bonding modification to straight polypeptide chains, causing a change in their structural shapes, with the end result allowing these proteins to gain function. Such protein functionality imbues in fungi, for example, virulence and pathogenicity [141]. Other examples of PTMs can be observed in proteins found in the cell membrane responsible for cell-to-cell interactions or secretory functions, which is extracellularly useful [142]. PTMs may be reversible or irreversible; however, external factors may cause irreversible changes to already modified proteins, which inevitably affects their ability to function as well. It must be noted that structure and shape are very much linked to specificity and the type of biochemical interactions individual proteins can participate in.

Wang et al. [143] describe filamentous fungi as having a mature PTMs machinery, especially for glycosylation. Unlike yeasts that mainly produce glycoproteins with high mannose content, filamentous fungi use many other monosaccharides for their glycosylation processes during PTMs, making them more appropriate to produce mammalian-like proteins for pharmaceutical uses. In their work, Wang et al. [144] reviewed the evolution of glycosylation in eukaryotes. The authors differentiate between N- and O-glycosylation. It should be noted that while N-glycoproteins have glycans attached to the amide group of their asparagine residues, O-glycoproteins have this modification attached to the carboxyl group of their serine, lysine, threonine, and proline residues. Additionally, they describe glycosylation as one of the most complex PTMs, but they assert that the process is crucial in excretory proteins’ folding. This assertion is confirmed by Ramazi and Zahiri [142], who remarkably linked glycosylation, or the lack thereof, to the onset of conditions such as cancer and diabetes in humans. Previously, Goto et al. [145] had reviewed structures and functions of O-glycosylation in fungi, providing a dossier of its cellular usefulness. Most fungal secretory proteins are glycosylated through the action of O-mannosyltransferase and several other glycosyltransferases as they move from the endoplasmic reticulum to the golgi apparatus before reaching the cell exterior. These modifications have been reported to add stability and solubility to extracellular proteins [142,145,146]. PTMs therefore play an important role in enzyme production strategies, especially when the research progression strategies may involve cloning and/or artificial synthesis. In this regard, Tokmakov et al. [147] investigated the correlation between PTMs and the success of heterologous protein synthesis. Results suggested that prior identification of potential PTMs using protein sequences could predict and optimise heterologous synthesis. Proteomics technologies such as mass spectrometry (MS) are able to study these modifications with the goal of identifying their cellular location, and the resulting data are stored on different databases, including the more popularly used PhosphoGRID, PHOSIDA, PhosphoELM, and iPTMNet [133,148].

Filamentous fungi have the advantageous tendency of producing extracellular enzymes, which allow them to play their role of complex compound degradation in the environment. This has largely contributed to extracellular enzymes being desirous in industrial applications because of the minimal requirement of downstream processing for their collection [12,149,150]. Recently, Arnau et al. [151] presented strategies and challenges for the production of industrial enzymes using the extracellular machinery of fungi. The authors described the use of classical mutagenesis and screening in order to identify and/or develop mutant organisms with the ability to produce higher enzyme titres. Moreover, many other strategies, including the use of antagonistic interspecific interactions, stronger promoters, codon optimisation of gene sequences, deletion of protease-coding genes, addition of artificial transcription factors, etc., have been employed for industrial enzyme production [152,153,154].

### 5.2. Enzyme Production through Recombinant DNA Technology

Cellular energy conservation and utilisation efficiency often imply that enzyme syntheses have feedback mechanisms and various other factors that prevent excessive production of all metabolites, including enzymes. Moreover, some organisms that are capable of enzyme over-expression may not necessarily be amenable to manageable growth conditions in the laboratory. Sometimes, the feasible solution to these challenges is the application of recombinant technology, involving the modification of a more amenable organism by integrating DNA molecules of the desired traits to allow its over-expression in the host organism, achieved through the use of a vector [155]. Since the introduction of recombinant DNA technology in the 1970s, it has contributed greatly to the large-scale production of many important proteins, including enzymes, in fields ranging from agriculture to pharmaceuticals and cosmetics [156,157]. Over the years, different expression hosts have been developed for recombinant protein production. In their work, Tripathi and Shrivastava [158] review these expression hosts, including bacteria, mammalian cells, yeasts, insects, and transgenic plants. The authors also point out the importance of using eukaryotic hosts to produce therapeutic proteins, as these require post-translational modifications, especially glycosylation, for their efficacy. As previously discussed, PTMs play a crucial role in eukaryotic protein stability, solubility, and functionality. As such, PTMs must therefore be considered during the selection of the host for recombinant protein production. Von Schaewen et al. [159] further explain this attribute as they discuss the limitations of bacterial hosts such as *E. coli* for the production of recombinant eukaryotic proteins.

Besides host selection, recombinant protein production also relies greatly on the effectiveness of cloning. The expression of recombinant eukaryotic proteins involves cloning of the cDNA of interest into an appropriate expression vector, followed by its insertion into the host cell [156]. Several cloning vectors are available depending on the intended use. Various cloning methods have also been developed. Jia and Jeon [160] describe different cloning methods and their possible applications for high-throughput recombinant protein production. These include restriction enzyme-based cloning, which utilises restriction enzymes to determine and cut the beginning and the end of the inserted gene; recombination-based cloning, where a site-specific recombinase is used to make the recombinant vector without using restriction enzymes; and ligation-independent cloning, which facilitates directional cloning of any insert with no restriction enzyme, nor recombinase needed. Additionally, Nishikawa et al. [161] provided an in-depth analysis of a group of plasmid vectors used for gene manipulation in fungi collectively referred to as ‘pFungiway’. Interestingly, the core constitution of this group of vectors is the binary plasmid pCAMBIA2200, and they advised that the vector *Agrobacterium tumefaciens* can be used to mediate transformation. They were able to use it to successfully bring about transformation in two fungi, a basidiomycete, *Flammulina velutipes*, and in the pathogenic plant fungus *Fusarium oxysporum*.

Different vectors can be designed to introduce a recombinant gene into the selected host. Depending on the chosen host, these vectors contain elements or motifs necessary for the optimal expression of the recombinant protein. These include a promoter region, affinity tags, fluorescence tag, and many more as described by previous researchers [162,163]. Various authors have reported different vectors compatible with eukaryotic hosts. Some of the most commonly used vectors for fungi in recent times are summarised in Table 2.

### 5.3. Expression Analysis

Depending on the objective of a study, protein expression analysis can be completed using high-throughput methods such as MS or protein arrays technology. Alharbi [172] is of the opinion that protein array is less preferred in comparison to MS due to the huge amounts of data it generates, making its application difficult for most studies. Moreover, its dynamic range of detection is limited as a result of both background and saturation levels signals and the interferences that they pose. Its potential to analyse hundreds of antibodies–antigen interactions at once brings with it the challenge of quantifying protein concentrations of the newly introduced proteins. It follows that it would need to possess the capability not only for detection but immediate quantification of the varying concentrations. It also has the additional problem of generating false-negative results in the basic investigation of protein interactions on an array. This can be a consequence of the inevitable change in the folding of a protein structure within the microarray, thereby destroying the antibody–antigen pairing, leading to a false-negative result. The same will likely occur in investigative analysis involving enzyme–substrate interactions, as the issue is associated with space constraints. Further, these challenges are possibly linked to the strategies employed in protein microarray detection, which are either label-based or label-free. The labelling process and its use of materials such as fluorescent dyes or radioisotopes tend to alter surface characteristics of the protein and also likely introduce biases to the results. Thus, there is a favouring of the label-free approach, which uses inherent properties of the probe samples such as its mass to avoid interference to the protein molecule within the probe. For example, in the application of surface plasmon resonance (SPR) as a technique, where proteins are immobilised on the microarray using a thin layer of gold coating on the surface structure when the unlabelled protein probe is added, any changes in the angle of reflection of light are regarded to have been caused by their interaction and used for detection. However, the sensitivity and specificity in the unlabelled approach are lower as compared to the label-based approach. It is hoped that with refinements in aspects of quantitative information on protein-binding kinetics, there will be a shift towards greater use of label-free approach [173].

On the other hand, the MS application brings with it accuracy and sensitivity, the two features that have contributed to its popularity. Data obtained through analysis by LC-MS/MS, MALDI-TOF/MS, for example, enable the discovery and identification of protein biomarkers, most of which can be found on databases such as Mascot, MS-Tag, and PepProb. In certain cases, selected proteins can be analysed using low-throughput techniques such as ELISA and Western blotting, which depend on the reaction between a protein and a complementary tag [148,172]. Previously, Braitbard and colleagues [174] used ELISA to assay human proteins using specific peptides and antibodies. These techniques not only confirm the presence or absence of a given protein, but they also, to some extent, give a quantitative proportionality to the total protein content of a sample. Although not common in fungal studies, ELISA can be effective for the serological detection of fungi; in this regard, some of its first applications go back to the 1990s with works such as that of Kim et al. [175], where ELISA was used to identify white- and brown-rot fungi with their ligninolytic metabolites serving as antigens. In more recent work, Martin-Souto et al. [176] used ELISA to detect fungi from cystic fibrosis patients. Using whole protein extract from *S. boydii* as an antigenic extract, *Scedosporium* and *Lomentospora* fungal species were serologically detected in patients’ sera with 100% sensitivity. Similar work has also been completed using Western blotting to identify fungi, but mostly those causing diseases [177]. More research should shift towards possible modifications and adaptations of these existing methodologies as they can be employed as a benchmark for developing methods more suited to research involving enzyme elucidation targeting industrial and environmental applications.

### 5.4. Protein (Enzyme) Purification and Separation

As many different fungal proteins and mostly enzymes are produced extracellularly, purification is necessary to separate the enzyme(s) of interest from the rest of the secretome. Purification is a prerequisite for most analytical work on proteins. Pure proteins are crucial for the effectiveness and accuracy of subsequent studies such as structural and functional determination [178]. Moreover, pure proteins are important for applications in various industries as the presence of contaminants could result in different and unwanted reactions. In the pharmaceutical industry, for instance, protein therapeutics require high levels of purity to prevent unwanted interferences and reactions that may result in human fatalities.

Purification aims to exploit differentiating physicochemical characteristics of enzymes in order to separate them from a mixture [179]. For example, Landi and colleagues [61,62] exploited this understanding in the purification of the novel protein Ageritin by applying acid precipitation techniques followed by chromatographic separation steps. As basic proteins, the high isoelectric point of ribotoxin-like proteins (≥9.5) allows them to remain soluble under acidic conditions as most contaminants precipitate.

Various techniques have been applied for protein purification, as summarised in Table 3 with most of them using liquid chromatography. More et al. [180] reported the chromatographic purification of a laccase enzyme using anionic exchange followed by gel filtration by means of Fast Protein Liquid Chromatography (FPLC). Using this approach, purification is initially executed based on the net charge of the proteins, followed by their molecular weights. Additionally, their study implemented an ultrafiltration step that used the Amicon^®^ system for further separation. This additional step enabled the concentration of the pure protein. In their studies, Mukhopadhyay et al. [181], Irfan et al. [182], and Carrasco et al. [183] used ammonium sulfate precipitation based on the biochemical process of salting in/out, which allows proteins to precipitate out of solution as a result of a change in ammonium sulfate ionic strength. When dealing with LMEs, careful considerations should be given to the material composition of filter panels; it is advisable to avoid reactive materials such as cellulose-based filters.

Regardless of the method used, purification is most often coupled with a separation technique for monitoring and evaluating the extent of purity. Cruz et al. [148] report that gel-based techniques remain the main separation techniques for proteins. Sodium dodecyl sulfate polyacrylamide gel electrophoresis (SDS-PAGE), two-dimensional gel electrophoresis (2-DE), and two-dimensional differential gel electrophoresis (2D-DIGE) allow separation and visualisation of proteins within a mixture. Furthermore, 2-DE gives the protein profile of a sample which is of valuable use in comparative studies using databases such as the World-2DPAGE [191,192].

### 5.5. Structural Studies and Sequencing

After purification, especially when working with a novel protein, structural studies are critical towards function predictions [193]. X-ray crystallography and NMR are the principal techniques used to determine the 3D structure of proteins [148,194]. Although they can be used individually, Yee et al. [194] describe these techniques as being complementary. This is also shown in the work of Bryn Fenwick et al. [195], where using these techniques in synergy gives more accuracy in the structural analysis. The structures derived from these studies can be deposited in the different structure databases such as Protein Data Bank (PDB), PDBsum, or ModBase [196]. Functions can be predicted using the various approaches reviewed by Mills et al. [197]. They provided notable perspectives on functional annotations at the molecular level of uncharacterised proteins but also highlighted 3D-structure-based methods for protein function prediction, which such studies benefit from using software programs such as MolScript. The analysis and determination of structure allow for protein sequence elucidation. Miyashita et al. [198] describe protein sequencing using Edman degradation, a classical technique used to determine the amino acid sequence of proteins. Although effective in many cases, Edman degradation is limited to proteins without post-translational modifications on the N-terminal. This limitation is the reason for the focus on mass spectrometry application as the more favoured approach in protein sequencing technology, even though more novel approaches are continuously being developed [199,200]. Using the sequences derived, bioinformatics tools such as UnitProt and RefSeq can be employed to develop a clearer perspective of the protein and its attributes [201]. However, the one feature that integrates a consortium of databases makes UniProt very popular as a platform for protein sequences. Its constituent databases, UniProt Knowledgebase, UniProt Archive, UniProt Reference Clusters, and UniProt Proteomes, cater to all protein inquiries from functional information, annotations, publications, homology, and full proteome information [201]. Similarly, RefSeq gives non-redundant sequence information of proteins, including conserved regions and variations. It is important to note that as part of NCBI, RefSeq can be accessed from all NCBI tools such as BLAST [202]. McGarvey et al. [203] describe the functioning of Refseq as it relates to the mouse genome annotations. The authors present the various resources accessible through Refseq, including gene annotations, publications, nucleotide and protein records, even including new features such as the ‘Identical Proteins Report’, which reveals proteins that are identical both in length and in sequence. It is a useful study for fungal protein elucidation from a eukaryotic classification standpoint.

## 6. Conclusions

The introduction of omics technologies has and continues to deepen our understanding of fungi and processes involved in the production of their unique and very useful enzymes. While molecular characterisation is considered a routine and necessary procedure, cohesion and sharing of information among the different available databases would provide better identification of new fungi species and reduce redundancy in research. Moreover, innovative adaptation of existing technologies designed for medical studies may be necessary to advance enzyme and protein studies targeting the industrial and environmental sector requirements of bioproducts. The study of fungal transcriptomes has enabled us to look beyond the genome as a static concept and perceive it as a dynamic collection of genes whose expressions are dependent on several factors. Transcriptomics studies have brought to our knowledge numerous genes that impact protein production both directly and indirectly. While we continue to rely on technologies such as RNA-seq for the identification of these genes, more transcriptomics studies are necessary to further elucidate the molecular basis of protein production, especially in the case of the important LMEs. Fungal proteomics also continues to be a growing area of research with the goal of discovering enzymes and proteins that will be more impactful to industry and the environment. Finally, bioinformatics analyses have gained importance as tools that facilitate the amalgamation of functional prediction and commercial value addition in our quest for bioproducts; they are tools that will continue to carry great significance in the future of research targeting protein elucidation and applications.

## Figures and Tables

**Table 2:** Examples of Eukaryotic expression vectors compatible with fungi.

Host	Vector	References
Yeast	Yeast Episomal Shuttle Vectors (YEp type)	[164]
	Yeast Integrative Plasmids (YIps)	[165,166]
	Yeast Replicative Plasmids (YRps)	[167,168]
	Yeast Centromeric Plasmids (YCps)	[169]
	Yeast Artificial Chromosome (YAC)	[168,170,171]
*Agrobacterium tumefaciens*	pFungiway vectors ^1^	[161]

^1^ Several vector types within this group.

**Table 3:** Protein purification techniques.

Technique	Principle	References
Salting in/out	Exploiting protein solubility by increasing salt concentration in the solution	[184]
Dialysis	Using size exclusion to separate proteins from small molecules and ions that pass through a semi-permeable membrane	[185]
Gel filtration chromatography	Separation of proteins based on size	[186]
Ion-exchange chromatography	Separation of proteins based on their net charge	[187,188]
Affinity chromatography	Exploiting the affinity of proteins for given chemical groups to separate them	[189]
High-Pressure Liquid Chromatography (HPLC)	Can use different principles of column chromatography to separate proteins using high pressure to give better resolution	[190]
